# Epidemiology and Clinical Characteristics of Chronic Kidney Disease in Bihar: A Cross-Sectional Study From a Single Center

**DOI:** 10.7759/cureus.64719

**Published:** 2024-07-17

**Authors:** Prit P Singh, Prem S Patel, Amresh Krishna, Shefali Kuntal, Sanjay Kumar, Om Kumar

**Affiliations:** 1 Nephrology, Indira Gandhi Institute of Medical Sciences, Patna, IND; 2 Community Medicine, Indira Gandhi Institute of Medical Sciences, Patna, IND

**Keywords:** epidemiology, hypertension, diabetes, anemia, chronic kidney disease

## Abstract

Background

Chronic kidney disease (CKD) is a major public health concern globally, often co-occurring with type 2 diabetes (T2D), hypertension (HTN), and cardiovascular disorders (CVD), which complicate its management and exacerbate outcomes. This study aims to investigate the epidemiological and clinical characteristics of CKD in Bihar, a region often underrepresented in national data.

Methods

This cross-sectional observational study was conducted at the Department of Nephrology, Indira Gandhi Institute of Medical Sciences (IGIMS) in Patna, Bihar, India. A total of 2,534 adult patients of both sexes who consented to participate were included. We collected demographic and clinical data, calculated the estimated glomerular filtration rate using the CKD-Epidemiology (CKD-EPI) Collaboration creatinine equation, and classified CKD stages. Statistical analyses were performed using IBM SPSS Statistics for Windows, version 29.0.2.0 (IBM Corp., Armonk, NY).

Result

The majority of the study population was male (66.5%), with a significant number residing in rural areas (76.8%). The prevalent causes of CKD included HTN (41.2%), chronic tubulointerstitial nephritis (31.8%), and T2D (23.2%). Approximately one-third of patients were in the early stages (Stages 1 and 2) of CKD. A high prevalence of anemia was noted across all stages, increasing significantly with glomerular filtration rate (GFR) reduction. Treatment analysis showed suboptimal use of angiotensin-converting enzyme inhibitors (ACEi) or angiotensin receptor blockers (ARBs) and other standard treatments like diuretics and statins, especially among T2D patients.

Conclusion

Chronic kidney disease in Bihar affects predominantly young males and is associated with significant rural prevalence and comorbidities like T2D, HTN, and CVD. Our results highlight the need for improved management practices, especially in the use of ACEi/ARBs and erythropoiesis-stimulating agents, to slow GFR reduction. Further multicentric, community-based studies are recommended to provide a more comprehensive understanding of CKD in Bihar.

## Introduction

Chronic kidney disease (CKD) significantly contributes to morbidity and mortality, often coexisting with type 2 diabetes (T2D), hypertension (HTN), and cardiovascular disorders (CVD). These conditions interact, complicating management and worsening outcomes. Chronic kidney disease predicts poor quality of life, cognitive decline, frequent hospitalizations, and substantial healthcare costs [1]. Its global prevalence has increased by 29.3% from 1990 to 2017, making it a major public health concern [2, 3]. In 2017, an estimated 697.5 million people worldwide (9.1% prevalence) suffered from CKD, with India alone accounting for 115.1 million cases, the second highest after China (132.2 million). The growing burden of CKD has moved it to the 12^th^ leading cause of death globally in 2017, up from the 17^th^ in 1990, underscoring its escalating impact [3]. In India, deaths attributed to CKD rose from 0.59 million in 1990 to 1.18 million in 2016 [4]. In lower- and middle-income countries, including India, CKD is frequently linked to infectious diseases, glomerulonephritis, and the inappropriate use of nephrotoxic agents like non-steroidal anti-inflammatory drugs, certain antibiotics, and traditional remedies. The absence of a central CKD registry in India likely leads to underestimations of its true incidence and prevalence. Previous studies have examined CKD’s epidemiology and clinical features across India [5-9], yet none included data from Bihar. This study aims to fill that gap by assessing the epidemiological and clinical characteristics of CKD in Bihar.

## Materials and methods

Study design

We conducted this cross-sectional observational study at the Department of Nephrology, Indira Gandhi Institute of Medical Sciences (IGIMS) in Patna, Bihar, India, over 18 months. The institutional ethics committee of IGIMS approved the study (approval no. 178/IEC/IGIMS/2021, dated June 26, 2021). We included patients with CKD aged over 18 years from both sexes who consented to participate, recruited from July 2021 to December 2023. We excluded individuals under the age of 18, pregnant women, patients with acute kidney injury, malignancy, psychiatric illnesses, or those who refused consent. We reviewed the files of 3,127 CKD patients, of whom 2,534 patients were finally included in this study. The rest of the patients were excluded due to refusal of consent, incomplete baseline laboratory investigations hindering interpretation, or discrepancies in data recording.

Data collection and statistical analysis

At their initial visit, we recorded the patients' demographic and clinical parameters. We obtained laboratory data from the hospital’s informatics system and documented them in a Microsoft Excel spreadsheet (Microsoft Inc., Redmond, WA). At this institute, creatinine levels (in either serum or urine samples) were measured using the colorimetric modified Jaffe’s method. We calculated the estimated glomerular filtration rate (eGFR) using the CKD-Epidemiology (CKD-EPI) Collaboration creatinine equation [10]. Urine routine analysis and urine protein estimation data were retrieved. Urine albumin was assessed using the dye-binding bromocresol purple (BCP) method. The urine albumin-creatinine ratio (ACR) was then determined and used to classify albuminuria as follows: normal to mildly increased (A1; ACR < 30 mg/gram), moderately increased (A2; ACR 30-299 mg/gram), and severely increased (A3; >300 mg/gram). We defined CKD as abnormalities in kidney structure or function persisting for more than three months with health implications [10]. We classified CKD stages based on eGFR: Stage 1 (>90 ml/minute), Stage 2 (89-60 ml/minute), Stage 3a (59-45 ml/minute), Stage 3b (30-44 ml/minute), Stage 4 (29-15 ml/minute), and Stage 5 (<15 ml/minute). We defined anemia as hemoglobin levels below 13.0 g/dl in men and below 12.0 g/dl in women. The severity of anemia was categorized as follows: mild (hemoglobin concentration 10-12 grams/deciliter (13 grams/deciliter in males)), moderate (hemoglobin concentration < 10 to 8 grams/deciliter), and severe anemia (hemoglobin concentration < 8 grams/deciliter) [10]. We documented treatment details for each patient and analyzed all data using IBM SPSS Statistics for Windows, version 29.0.2.0 (IBM Corp., Armonk, NY) for further analysis. The normality of variables was checked by the Kolmogorov-Smirnov test. The results of non-parametric variables were presented as medians and interquartile ranges (IQRs). The Chi-square or Fisher's exact test was used to compare the categorical variables. The results of the Chi-square test were tabulated as frequency and percentage. A p-value <0.05 was considered statistically significant.

## Results

Our study enrolled 2,534 patients with CKD, predominantly male (n = 1,685; 66.5%), with a median age of 41 (IQR: 28 to 55 years). A majority (n = 1,947; 76.8%) of these patients lived in rural areas. The most common causes of CKD were HTN (n = 1,044; 41.2%), chronic tubulointerstitial nephritis (n = 806; 31.8%), and T2D (n = 589; 23.2%). Other significant contributors included polycystic kidney disease (n = 454; 17.9%) and chronic glomerulonephritis (n = 435; 17.2%). Coronary artery disease (CAD) and hypothyroidism were present in 296 (11.7%) and 108 (4.3%) patients, respectively. The median systolic and diastolic blood pressures were 130 mmHg (IQR: 120 to 140 mmHg) and 80 mmHg (IQR: 70 to 90 mmHg). Substance use was observed in 139 patients (5.5%), predominantly tobacco chewing (n = 106; 4.2%). A further 160 patients (6.3%) reported using indigenous (n = 55; 2.2%) or other nephrotoxic medications (n = 105; 4.1%). Table 1 presents a comprehensive breakdown of these characteristics.

**Table 1:** Baseline characteristics of the CKD study population (N = 2,534) CKD: chronic kidney disease; eGFR: estimated glomerular filtration rate

Characteristics		N (%)
Sex	Male	1685 (66.5)
	Female	849 (33.5)
Area of residence	Urban	587 (23.2)
	Rural	1947 (76.8)
Hypertension		1044 (41.2)
Chronic tubulointerstitial nephritis		806 (31.8)
Diabetes mellitus		589 (23.2)
Polycystic kidney disease		454 (17.9)
Chronic glomerulonephritis		435 (17.2)
Coronary artery disease		296 (11.7)
Hypothyroidism		108 (4.3)
Arthritis		88 (3.5)
eGFR (ml/min/1.73m^2^)	≥ 90	847 (33.4)
	60 – 89	846 (33.4)
	45 – 59	376 (14.8)
	30 – 44	123 (4.9)
	15 – 29	170 (6.7)
	<15	172 (6.7)
Anemia	Mild	891 (35.2)
	Moderate	765 (30.2)
	Severe	482 (19.0)
Substance use (addiction)	Tobacco chewing	106 (4.2)
	Alcohol	29 (1.1)
	Smoking	4 (0.2%)
Drug	Indigenous medication	55 (2.2)
	Nephrotoxic medications	105 (4.1)

Out of 2,534 patients, urine protein data was available for 2,142 patients, with a mean spot urine ACR of 260 mg/gram. Among these 2,142 patients, 838 (39%) had normal to mildly increased albuminuria (A1), 997 (46.6%) had moderately increased albuminuria (A2), and only 307 (14%) had severely increased albuminuria (A3). Among diabetic patients, the majority (204, 50.7%) had severely increased albuminuria, while 113 (28.1%) had moderately increased albuminuria, and 85 (21.1%) had normal to mildly increased albuminuria. Among non-diabetic patients, 753 (43.2%) had normal to mildly increased albuminuria, 884 (50.8%) had moderately increased albuminuria, and only 103 (5.9%) had severely increased albuminuria.

According to the Kidney Disease: Improving Global Outcomes (KDIGO) criteria, the distribution of CKD stages was fairly even, with 33.4% of patients in both Stages 1 (n = 847) and 2 (n = 846); 14.8% in Stage 3a (n = 376); 4.9% in Stage 3b (n = 123); and 6.7% in both Stages 4 (n = 170) and 5 (n = 172). A high proportion of patients (n = 2,138; 84.4%) had anemia, categorized as mild (n = 891; 35.2%), moderate (n = 765; 30.2%), or severe (n = 482; 19.0%). The prevalence of severe anemia increased notably with advancing CKD stages, with 106 (12.5%) in Stage 1, 129 (15.2%) in Stage 2, 113 (30%) in Stage 3a, 27 (22%) in Stage 3b, 40 (23.4%) in Stage 4, and 67 (40%) in Stage 5, as detailed in Table 2.

**Table 2:** Distribution of anemia and its severity among participants according to CKD stages (N = 2,138) CKD: chronic kidney disease; eGFR: estimated glomerular filtration rate

CKD Stages	Normal, n (%)	Mild, n (%)	Moderate, n (%)	Severe, n (%)	Total, N (%)
Stage 1 (eGFR >90 ml/min/1.73m^2^)	197 (23.2)	337 (49.7)	207 (24.4)	106 (12.5)	847 (100)
Stage 2 (eGFR 89-60 ml/min/1.73m^2^)	123 (14.5)	359 (42.4)	235 (27.7)	129 (15.2)	846 (100)
Stage 3a (eGFR 59-45 ml/min/1.73m^2^)	20 (5.4)	92 (24.5)	151 (40.1)	113 (30)	376 (100)
Stage 3b (eGFR 30-44 ml/min/1.73m^2^)	21 (17.1)	38 (30.9)	37 (30.)	27 (22)	123 (100)
Stage 4 (eGFR 29-15 ml/min/1.73m^2^)	15 (8.8)	44 (26)	71 (41.8)	40 (23.4)	170 (100)
Stage 5 (eGFR <15 ml/min/1.73m^2^)	20 (11.6)	21 (12.2)	64 (37.2)	67 (40)	172 (100)
Total	396 (16%)	891 (35%)	765 (30%)	482 (19%)	2534 (100)

Overall, HTN was present in 1,336 (52.7%) patients. Patients with T2D demonstrated a higher incidence of HTN (n = 379, 64.3% vs. n = 957, 49.2%; p<0.001) and CAD (n = 169, 28.7% vs. n = 127, 6.5%; p<0.001) than patients without T2D. They also showed a higher prevalence of anemia (n = 530, 90%) than patients without T2D (n = 1,608, 82.7%). These patients were more likely to present at advanced CKD stages, with significant differences observed in Stages 3 through 5 (n = 138, 23.5% vs. n = 361, 18.6% for Stage 3; p = 0.042). Table 3 presents detailed demographic comparisons and additional statistical data.

**Table 3:** Comparison of baseline demographic characteristics in patients with and without T2D (N = 2,534) T2D: type 2 diabetes; CAD: coronary artery disease; CKD: chronic kidney disease; HTN: hypertension *Chi-square test used

Characteristics		Total (N=2534), n (%)	Diabetes		P-value^*^
			Yes (N=589), n (%)	No (N=1945), n (%)	
Comorbidities	Hypothyroidism	108 (4.3)	28 (4.8)	80 (4.1)	<0.001
	CAD	296 (11.7)	169 (28.7)	127 (6.5)	
	HTN	876 (34.6)	319 (54.2)	557 (28.6)	
	None	1237 (48.8)	67 (11.4)	1170 (60.2)	
Gender	Male	1685 (66.5)	381 (64.7)	1304 (67.0)	0.28
	Female	849 (33.5)	208 (35.3)	641 (33.0)	
Anemia	Normal	396 (15.6)	59 (1.0)	337 (17.3)	<0.001
	Mild	891 (35.2)	201 (34.1)	690 (35.5)	
	Moderate	765 (30.2)	213 (36.2)	552 (28.4)	
	Severe	482 (19)	116 (19.7)	366 (18.8)	
Marital status	Married	2358 (93.1)	583 (99)	1775 (91.3)	<0.001
	Unmarried	176 (6.9)	6 (1)	170 (8.7)	
Education	Illiterate	927 (36.6)	248 (42.1)	679 (34.9)	<0.001
	Primary	332 (13.1)	58 (9.8)	274 (14.2)	
	Middle	177 (7.0)	42 (7.1)	135 (6.9)	
	High school	600 (23.7)	125 (21.2)	475 (24.4)	
	Intermediate/Diploma	186 (7.3)	52 (8.8)	134 (6.9)	
	Graduate	268 (10.6)	50 (8.5)	218 (11.2)	
	Professional	44 (1.7)	14 (2.4)	30 (1.5)	
Occupations	Unemployed	1784 (70.4)	433 (73.5)	1351 (69.5)	<0.001
	Unskilled	31 (1.2)	6 (1.0)	25 (1.3)	
	Semi-skilled	75 (3.0)	15 (2.5)	60 (3.1)	
	Skilled	50 (2.0)	8 (1.4)	42 (2.2)	
	Clerical/Shop	579 (22.8)	114 (19.4)	465 (23.9)	
	Semi-professional	7 (0.3)	7 (1.2)	0 (0)	
	Professional	8 (0.3)	6 (1)	2 (0.1)	
CKD stage	Stage 1 (eGFR>90 ml/min/1.73m^2^)	847 (33.4)	168 (28.5)	679 (34.9)	0.420
	Stage 2 (eGFR 89-60 ml/min/1.73m^2^)	846 (33.4)	198 (33.6)	648 (33.3)	
	Stage 3a (eGFR 59-45 ml/min/1.73m^2^)	376 (14.8)	104 (17.7)	272 (14.0)	
	Stage 3b (eGFR 44-30 ml/min/1.73m^2^)	123 (4.9)	34 (5.8)	89 (4.6)	
	Stage 4 (eGFR 30-15 ml/min/1.73m^2^)	170 (6.7)	40 (6.8)	130 (6.7)	
	Stage 5 (eGFR <15 ml/min/1.73m^2^)	172 (6.7)	45 (7.6)	127 (6.5)	

Among all participants, 494 (19.5%) were asymptomatic. The most prevalent symptoms were weakness (n = 704; 27.9%), uremic symptoms (n = 570; 22.5%), and abdominal pain (n = 220; 8.7%). The complete symptom distribution is presented in Table 4.

**Table 4:** Chief presenting symptoms in CKD patients (N = 2,534) CKD: chronic kidney disease; LUTS: lower urinary tract symptom

Symptoms	N (%)
Asymptomatic	494 (19.5)
Weakness	704 (27.9)
Uremic symptoms	570 (22.5)
Pain in the abdomen	220 (8.7)
Edema	198 (7.8)
Breathlessness	116 (4.6)
Frothuria	57 (2.2)
Headache	52 (2.1)
Macroscopic hematuria	34 (1.3)
Febrile illness	31 (1.2)
LUTS	31 (1.2)
Microscopic hematuria	25 (1.0)
Total	2534 (100)

The analysis of treatment prescriptions indicated that 698 (27.5%) patients were taking antihypertensive medications, of which 320 (12.6%) were on angiotensin-converting enzyme inhibitors (ACEi) or angiotensin receptor blockers (ARBs). Diuretics were used by 517 (20.4%) of patients, more commonly in T2D patients (n = 193, 32.7% vs. n = 324, 16.6%; p<0.001). Similarly, statins were prescribed to 1,019 patients (40.2%), again more frequently in patients with T2D (n = 430, 73% vs. n = 589, 30.2%; p<0.001). Phosphate binders and erythropoiesis-stimulating agents were also more commonly used in T2D patients (n = 62, 10.5% vs. n = 105, 5.4%, and n = 129, 21.9% vs. n = 209, 10.7%, respectively; p<0.001). Further details are presented in Table 5.

**Table 5:** Use of various medications in diabetic and non-diabetic CKD populations (N = 2,534) CKD: chronic kidney disease; ACEi: angiotensin-converting enzyme inhibitor; ARBs: angiotensin-II receptor blockers; ESA: erythropoiesis-stimulating agents *Chi-square test used

Characteristics		Total, n (%)	Diabetes		P-value^*^
			Yes (N=589), n (%)	No (N=1945), n (%)	
Anti-hypertensive	Yes	698 (27.5)	211 (35.8)	487 (25)	<0.001
	No	1836 (72.2)	378 (64.2)	1458 (75)	
ACEi/ARBs	Yes	320 (12.6)	124 (21.1)	196 (10.1)	<0.001
	No	2214 (87.4)	465 (78.9)	1749 (89.9)	
Statins	Yes	1019 (40.2)	430 (73)	589 (30.2)	<0.001
	No	1515 (59.8)	159 (27)	1356 (69.8)	
Diuretics	Yes	517 (20.4)	193 (32.7)	324 (16.65)	<0.001
	No	2017 (79.6)	396 (67.3)	1621 (83.35)	
Calcium supplements	Yes	849 (33.5)	272 (46.2)	577 (29.7)	<0.001
	No	1685 (66.5)	317 (53.8)	1368 (70.3)	
Phosphate binder	Yes	167 (6.6)	62 (10.5)	105 (5.4)	<0.001
	No	2367 (93.4)	527 (89.5)	1840 (94.6)	
Iron supplements	Yes	752 (29.7)	251 (42.6)	501 (25.8)	<0.001
	No	1782 (70.3)	338 (57.4)	1444 (74.2)	
Sodium bicarbonate	Yes	756 (29.8)	245 (41.6)	511 (26.3)	<0.001
	No	1778 (70.2)	344 (58.4)	1434 (73.7)	
ESA	Yes	338 (13.3)	129 (21.9)	209 (10.7)	<0.001
	No	2196 (86.7)	460 (78.1)	1736 (89.3)	

Our findings also highlighted differences in CKD prevalence between rural and urban areas. Anemia was more prevalent in the rural population (n = 1,682, 86.3% vs. n = 456, 77%). The majority of rural residents with CKD had an annual income of less than Rupees (Rs.) 100,000.00 (n = 872; 44.8%) or between Rs. 100,000.00 and Rs. 250,000.00 (n = 1,044; 53.6%), whereas urban residents more commonly earned between Rs. 100,000.00 and Rs. 250,000.00 (n = 350; 59.7%) or more than Rs. 250,000.00 (n = 68; 11.5%). Table 6 provides additional socioeconomic data.

**Table 6:** Characteristics of CKD patients living in rural and urban areas (N = 2,534) CKD: chronic kidney disease

Characteristics		Rural, N=1947 (76.8%), n (%)	Urban, N=587 (23.2%), n (%)
		Gender	Male	1248 (64)	437 (74.4)
Female	699 (36)		150 (25.6)
Education	Illiterate	859 (44)	68 (11.5)
	Primary	441 (22.6)	68 (11.5)
	High School	423 (21.7)	177 (30)
	Inter and Above	224 (11.5)	274 (47)
Anemia	Mild	672 (40)	219 (48)
	Moderate	621 (37)	144 (31.6)
	Severe	389 (23)	93 (20.4)
Income (lakh per annum)	<100,000	872 (44.8)	169 (28.8)
	100,000-250,000	1044 (53.6)	350 (59.7)
	≥250,000	31 (1.6)	68 (11.5)

Of the 106 patients undergoing dialysis, 73 (68.9%) used non-tunneled catheters, 19 (17.9%) used tunneled temporary catheters, and 14 (13.2%) had an arteriovenous fistula. Dialysis frequency varied, with 61 (57.5%) receiving bi-weekly treatments, 29 (27.3%) dialyzing irregularly, and 12 (11.3%) undergoing maintenance hemodialysis three times per week.

## Discussion

This hospital-based cross-sectional study investigated the prevalence, epidemiology, demographic characteristics, comorbidities, clinical staging, management, and prescription patterns of CKD in Bihar, one of India’s poorer states with suboptimal health indices [11]. We enrolled 2,534 participants, with a median age of 41 years (IQR: 28 to 55 years). This age is comparable to findings from other Indian studies: 41.4 ± 12.7 years in Chennai, 44.4 ± 13.9 years in Delhi, and 50.1 ± 14.6 years in the Center for Cardiometabolic Risk Reduction in South Asia (CARRS) surveillance study by Anand et al. [12]. The male-to-female ratio in our study was 1.98, similar to the 2.3 ratio reported in the Indian CKD registry [7]. Nayak-Rao in Assam found 70% male prevalence in a cohort of 334 CKD patients [13], while the CARRS surveillance study observed near equal or slightly higher CKD prevalence in females (7.5% vs. 7.7%) [12]. These variations in gender distribution may be attributable to occupational differences, a higher likelihood of males seeking treatment, and societal gender biases in healthcare access in Bihar. In patriarchal societies, where men often serve as primary earners, their health tends to be prioritized.

Our study found that 76.8% of patients lived in rural areas, and 63.6% had received a formal education, aligning with findings from the Indian Chronic Kidney Disease (ICKD) Phase 1 study, where approximately two-thirds of patients were from rural areas and approximately 73% were educated [9]. The literacy rate in Bihar is similarly reported at 61.8% [14]. We observed socioeconomic disparities between rural and urban CKD patients: rural patients typically had lower education levels, were often employed in unskilled or semiskilled physical labor, and earned less. Urban CKD patients, while generally more educated, also faced unemployment and lower earnings. These findings are echoed in the ICKD study, which noted higher illiteracy rates among rural CKD patients (33.21% vs. 14.6% in urban areas) and higher annual incomes among urban residents [9].

Our findings suggest that CKD is prevalent among individuals with lower socioeconomic status, regardless of geographical location. This trend could be linked to limited access to healthcare facilities in remote rural areas, driven by both a lack of awareness and affordability. The ICKD study indicated no significant difference in the prevalence of comorbidities such as T2D, HTN, or CVD [9]. This uniformity implies that many patients present at later stages of the disease due to the distance from tertiary healthcare facilities and the high treatment costs, which are predominantly out-of-pocket in India. Rajapurkar et al. also noted that insufficient health facilities and awareness often prevent early diagnosis of CKD, leading to patients from rural areas presenting at more advanced stages, which increases treatment costs and worsens the overall prognosis [7]. In our study, the majority (n = 1,380, 85.6%) of patients had mild (A1) to moderate (A2) levels of albuminuria, while only 307 (14%) had severely increased albuminuria (A3). Among diabetic patients, the majority (204, 50.7%) had severely increased albuminuria, whereas the majority (n = 1637, 94%) of non-diabetic patients had mild (A1) to moderate (A2) levels of albuminuria. Our observation supports the notion that diabetic patients present with albuminuria early in the course of the disease.

In our study, two-thirds of the patients were diagnosed with early CKD (Stages 1 or 2). This classification was based on including proteinuria of more than three months as a diagnostic criterion for CKD, similar to the approach used in the Screening and Early Evaluation of Kidney Disease (SEEK) study [8]. In contrast, a study from Chennai defined CKD using an eGFR cut-off of less than 80 ml/minute [5], and Agarwal et al. in Delhi used a serum creatinine threshold greater than 1.8 mg/dL [6]. Since serum creatinine and creatinine-based equations can be unreliable in accurately measuring kidney damage, these studies likely underestimated the number of patients with early-stage CKD who exhibited only proteinuria with normal serum creatinine levels. According to the KDIGO guidelines, such patients should be classified as having CKD [15].

Variations in the definitions and inclusion criteria for CKD across different studies have led to disparate prevalence rates. Our study identified the following prevalence rates for CKD stages: 33.4% for Stages 1 and 2, 19.7% for Stage 3, and 6.7% for Stages 4 and 5. These rates align closely with the distribution reported in the SEEK study, which documented a stage prevalence of 7:4.3:4.3:0.8:0.8 [8]. Anand et al. and Varma et al. also noted a high prevalence of early-stage CKD, with 80% and double the proportion of patients in Stages 1 and 2 compared to Stage 3, respectively [12,16]. Conversely, Rai et al. reported a lower prevalence of early CKD, with 5.8%, 4.9%, and 6.8% for Stages 1, 2, and 3, respectively [17].

When analyzing CKD patients by diabetic status, 589 (23.2%) had T2D, and the majority, 1,945 (76.8%), were non-diabetic. A larger proportion of T2D patients were in the higher stages of CKD compared to non-diabetic patients (n = 223, 38% vs. n = 618, 32%). Specifically, 38% of T2D patients were distributed as follows: 104 (17.7%) in Stage 3a, 34 (5.8%) in Stage 3b, 40 (6.8%) in Stage 4, and 45 (7.6%) in Stage 5. These disparities may be attributable to differences in inclusion criteria and a lack of awareness among the population and local practitioners about CKD, resulting in delayed evaluation of kidney function. Similar trends of more advanced disease and severe anemia in T2D patients have been reported in other studies [12,18].

The prevalence of HTN in our study was significantly lower than that reported in the ICKD study and by Rao et al. [9,13], but it was similar to figures from the Indian CKD registry and the CARRS registry [7,12]. We have compared the baseline characteristics of our study with those of other national and international CKD registries in Table 7 [7-9,19,20].

**Table 7:** Basic clinical and epidemiological characteristics of major CKD registries CKD: chronic kidney disease; T2D: type 2 diabetes; CVD: cardiovascular disease; DKD: diabetic kidney disease; HTN: hypertension; CGN: chronic glomerulonephritis; CIN: chronic interstitial nephritis; PKD: polycystic kidney disease; eGFR: estimated glomerular filtration rate; SEEK: Screening and Early Evaluation of Kidney Disease; ICKD: Indian Chronic Kidney Disease; KNOW-CKD: KoreaN Cohort Study for Outcomes in Patients With Chronic Kidney Disease; CRIC: Chronic Renal Insufficiency Cohort; NA: not applicable

Characteristics	Present Study (n=2534)	Indian CKD [7] (n= 52,273)	SEEK [8] (n= 5588)	ICKD [9] (n=4056)	KNOW-CKD [19] (n=2,238)	CRIC [20] (n=3612)
Age (years)	42.13±16.85	50.1 ± 14.6	45.22 ± 15.2	50.3 ± 11.8	53.7 ±12.2	58.2 ± 11
Female (%)	33.5	29.7	44.9	32.8	38.8	46
Mean eGFR +SD (mL/min/ 1.73m^2^)	43.2 ± 37.3	NA	NA	40.6 ± 17.2	53.1 ± 30.7	43.4 ±1 3.5
T2D (%)	37.7	33.1	18.8	37.5	33.7	47
HTN (%)	52.7%	NA	43.1	87	96.1	86
CVD (%)	11.7	NA	7	21.8	6	33
Major causes of CKD (%)	DKD (23.2)	DKD (31.3)	DKD (18.8)	DKD (24.9)	DKD (23.2)	DKD (25.5)
	HTN (34.5)	HTN (12.9)	HTN (7.9)	HTN (7.9)	HTN (18.3)	HTN (14.5)
	CGN (17.2)	CGN (13.8)	NA	CGN (14.7)	CGN (36.2)	NA
	CIN (31.8)	NA	NA	CIN (23.2)	NA	NA
	PKD (17.9)	PKD (2.6)	NA	NA	PKD (16.3)	NA

Similar to findings from the Indian CKD registry and SEEK study [8], approximately two-thirds of our CKD patients had an annual income of less than Rs. 100,000. Bihar’s per capita income in the financial year 2022-23 was Rs. 54,000, notably lower than the national average of Rs. 172,000, making it the state with the lowest per capita income among larger Indian states [21]. Consequently, financial constraints may impede access to healthcare, leading to a higher prevalence of CKD, delayed diagnoses, and progression to the advanced stages of the disease.

In our cohort, only 320 (12.6%) patients were prescribed ACEi or ARBs, with T2D patients twice as likely to receive these medications compared to non-diabetic patients. This contrasts with the 20.5% reported by Tuttle et al. [22]. Additionally, statins were prescribed to 1,019 (40.2%) of our patients, which differs from the 17.7% reported in Tuttle et al.’s study. These discrepancies highlight a gap in the optimal management of CKD, underscoring the need to promote the use of ACEi/ARBs to slow GFR reduction. The prescription of statins, particularly in T2D and CAD patients, reflects standard practice.

Our study also observed nephrolithiasis in 256 (10.1%) patients, with 60% experiencing bilateral obstruction which is higher than the 5.2% prevalence reported in the SEEK study [8]. This suggests a potential link between bilateral renal calculi and CKD progression in both T2D and non-diabetic patients. Typically, unilateral renal calculi present with renal colic and normal renal function, leading patients primarily to urology outpatient departments.

The most common symptoms in our cohort were weakness (n = 704; 27.9%) and uremic symptoms such as anorexia, nausea, and vomiting (n = 570; 22.5%). Four hundred ninety-four patients (19.5%) were asymptomatic, discovered through incidental findings of proteinuria or other laboratory abnormalities. Galhotra et al. reported higher frequencies of these symptoms [23].

Anemia was prevalent in 2,138 (84.4%) of our patients, with a mean hemoglobin concentration of 8.42 ± 2.2 g/dL. Severe anemia (hemoglobin concentration <8 g/dL) was more common and severe in Stage 5 CKD compared to Stage 3. The proportion of severe anemia was significantly higher in Stage 5 (n = 67; 40%) than in Stages 1 and 2 (n = 106, 12.5%, and n = 129, 15.2%, respectively), aligning with US Renal Data System 2023 data [23]. The high prevalence of anemia may reflect underlying malnutrition and iron deficiency, particularly in economically disadvantaged rural areas. Only 338 (13.4%) anemic patients received erythropoiesis-stimulating agents, and a mere 165 (6.5%) received blood transfusions, indicating inadequate treatment for anemia. This low transfusion rate could be attributed to Bihar's scarcity of blood donations, compounded by myths about post-transfusion weakness and a general reluctance to engage with health services.

Regarding dialysis, among 106 Stage 5 CKD patients, only 14 (13%) had arteriovenous fistulas for hemodialysis, with the remainder using temporary non-tunneled internal jugular catheters. Twenty-nine patients (27%) did not receive regular dialysis due to the high demand and limited capacity at government centers, leading to inadequate treatment and severe outcomes. Bihar’s shortage of nephrology services is exacerbated by the fact that only approximately 25 nephrologists are available for a population of 120 million, predominantly in urban areas.

Our study had several important limitations. Being a hospital-based study, the findings cannot be extrapolated to the general population. Despite being conducted at the largest government tertiary care institute in Bihar, which attracts a substantial patient influx from neighboring states and even Nepal, the results may not apply universally to other centers. Additionally, as IGIMS is a public sector hospital, it may disproportionately represent patients from lower socioeconomic strata, potentially not reflecting the condition of the more affluent segments of society. These factors limit the generalizability of our findings and suggest the need for caution in interpreting the scope of CKD prevalence and management practices derived from this single-center study.

## Conclusions

This study aimed to examine the epidemiological and clinical features of CKD in Bihar, a region frequently overlooked in national data. Our study observed that CKD patients in Bihar are predominantly young males, with a significant proportion residing in rural areas characterized by low literacy and income levels. Common comorbidities among these patients included T2D, HTN, and CAD, and they often presented at the early stages of CKD. Anemia was more prevalent and tended to be more severe in the advanced stages of the disease. The underuse of ACEi, ARBs, and erythropoiesis-stimulating agents underscores a critical area for improvement in clinical practice. To gain a deeper understanding of the prevalence, etiology, and outcomes of CKD in this region, conducting a larger, multicentric, community-based study with long-term follow-up is essential.

## References

[1] Hill NR, Fatoba ST, Oke JL, Hirst JA, O'Callaghan CA, Lasserson DS, Hobbs FD (2016). Global prevalence of chronic kidney disease - a systematic review and meta-analysis. PLoS One.

[2] Sundström J, Bodegard J, Bollmann A (2022). Prevalence, outcomes, and cost of chronic kidney disease in a contemporary population of 2·4 million patients from 11 countries: The CaReMe CKD study. Lancet Reg Health Eur.

[3] (2020). Global, regional, and national burden of chronic kidney disease, 1990-2017: a systematic analysis for the Global Burden of Disease Study 2017. Lancet.

[4] Xie Y, Bowe B, Mokdad AH (2018). Analysis of the Global Burden of Disease study highlights the global, regional, and national trends of chronic kidney disease epidemiology from 1990 to 2016. Kidney Int.

[5] Mani MK (2005). Experience with a program for prevention of chronic renal failure in India. Kidney Int Suppl.

[6] Agarwal SK, Dash SC, Irshad M, Raju S, Singh R, Pandey RM (2005). Prevalence of chronic renal failure in adults in Delhi, India. Nephrol Dial Transplant.

[7] Rajapurkar MM, John GT, Kirpalani AL (2012). What do we know about chronic kidney disease in India: first report of the Indian CKD registry. BMC Nephrol.

[8] Singh AK, Farag YM, Mittal BV (2013). Epidemiology and risk factors of chronic kidney disease in India - results from the SEEK (Screening and Early Evaluation of Kidney Disease) study. BMC Nephrol.

[9] Kumar V, Yadav AK, Sethi J (2022). The Indian Chronic Kidney Disease (ICKD) study: baseline characteristics. Clin Kidney J.

[10] Kidney Disease: Improving Global Outcomes (KDIGO) CKD Work Group (2024). KDIGO 2024 clinical practice guideline for the evaluation and management of chronic kidney disease. Kidney Int.

[11] (2024). National health profile. https://cbhidghs.nic.in/index1.php.

[12] Anand S, Shivashankar R, Ali MK (2015). Prevalence of chronic kidney disease in two major Indian cities and projections for associated cardiovascular disease. Kidney Int.

[13] Nayak-Rao S (2024). Profile of chronic kidney disease from a nephrology underserviced region in North Eastern India: a preliminary report from a single center in Assam. Int Urol Nephrol.

[14] (2024). Bihar literacy rate. https://www.indiacensus.net/states/bihar/literacy.

[15] Varma PP (2015). Prevalence of chronic kidney disease in India - where are we heading?. Indian J Nephrol.

[16] Rai PK, Rai P, Bedi S (2019). Prevalence and risk factors of chronic kidney disease in overweight and obese population in a tertiary care hospital in North India. Saudi J Kidney Dis Transpl.

[17] Iwai T, Miyazaki M, Yamada G (2018). Diabetes mellitus as a cause or comorbidity of chronic kidney disease and its outcomes: the Gonryo study. Clin Exp Nephrol.

[18] (2024). Per capital income across Bihar in Indian from financial year 2012 to 2023. https://www.statista.com/statistics/1117488/india-per-capita-income-bihar/.

[19] Oh KH, Kang M, Kang E (2020). The KNOW-CKD Study: what we have learned about chronic kidney diseases. Kidney Res Clin Pract.

[20] Lash JP, Go AS, Appel LJ (2009). Chronic Renal Insufficiency Cohort (CRIC) Study: baseline characteristics and associations with kidney function. Clin J Am Soc Nephrol.

[21] Tuttle KR, Alicic RZ, Duru OK (2019). Clinical characteristics of and risk factors for chronic kidney disease among adults and children: an analysis of the CURE-CKD Registry. JAMA Netw Open.

[22] Galhotra A, Rathore V, Pal R (2023). Clinico-epidemiological profile of patients with chronic kidney diseases of unknown etiology: a hospital-based, cross-sectional study from central India. Indian J Nephrol.

[23] Johansen KL, Gilbertson DT, Li S (2024). US Renal Data System 2023 Annual Data Report: epidemiology of kidney disease in the United States. Am J Kidney Dis.

